# ManuTrace: An HTML‐Based Interface for Manually Extracting Movement Trajectory Data From a Video

**DOI:** 10.1002/ece3.73804

**Published:** 2026-06-08

**Authors:** Kiyotaka Yabe, Yuna Tosaka, Kenji Matsuura

**Affiliations:** ^1^ Laboratory of Insect Ecology, Graduate School of Agriculture Kyoto University Kyoto Japan; ^2^ Distinguished Doctoral Program of Platforms Kyoto University Kyoto Japan

**Keywords:** animal movement, behavioral ecology, software, tracking, trajectory extraction, video analysis

## Abstract

Tracking the movements of animals provides fundamental insights into behavioral strategies and ecological adaptations. Recent advances in automated video analysis have enabled the extraction of movement trajectories from video recordings. However, effective automated tracking often requires careful adjustment of recording environments and iterative tuning of software parameters, imposing substantial costs in user proficiency and trial‐and‐error processes before analyzable data can be obtained. In exploratory analyses of pilot or opportunistically recorded videos, rapid extraction of trajectories from a limited number of videos is often more important than throughput, making such setup costs a practical barrier. Here, we introduce ManuTrace, an HTML‐based interface for manual extraction of trajectory data from videos. In this tool, users interactively play, pause, and seek videos and record object positions by mouse clicks, with positions in intermediate frames automatically interpolated and the resulting trajectories exported as CSV files for downstream analysis. As a demonstration, we extracted trajectories from videos of termite groups recorded under both natural and tracking‐optimized conditions. Automated tools showed highly variable tracking accuracy depending on recording conditions and individual behavior, often requiring substantial manual correction under unfavorable conditions. In contrast, ManuTrace enabled consistent trajectory extraction across recording conditions, processing a 1800‐frame video with 12 individuals (21,600 coordinates in total) within 20 min. The software consists of a single HTML file and runs in any modern web browser without installation, allowing use across diverse environments. Owing to its simplicity and portability, ManuTrace complements automated tracking tools as an entry point to trajectory analysis and also provides a useful resource for educational purposes.

## Graphical abstract

ManuTrace is a lightweight, browser‐based tool for manually extracting movement trajectories from video recordings. Designed to minimize technical setup, it provides an intuitive interface for annotating and visualizing trajectories. ManuTrace complements automated tracking approaches and is particularly suited to exploratory analyses and pilot studies, where reliable trajectory data must be obtained consistently from small datasets under variable recording environments without extensive technical optimization.

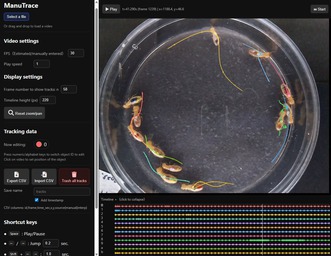

## 1 Introduction

Movement trajectories reflect how individuals search for resources (Bell 1990; Kareiva and Shigesada 1983; Pyke et al. 1977), avoid threats (Dill and Houtman 1989; Lima and Dill 1990), and explore their surroundings (Benhamou 2004; Denenberg 1969). In social or group‐living species, particularly, trajectories of multiple individuals reveal patterns of interaction, coordination, and collective organization (Ballerini et al. 2008; Buhl et al. 2006; Couzin et al. 2005; Partridge 1982). Consequently, the extraction and analysis of movement trajectories from video recordings has become a standard and powerful approach in behavioral ecology and related fields (Anderson and Perona 2014; Brown and De Bivort 2018; Dell et al. 2014).

Recent advances in video‐based tracking have been driven primarily by developments in machine learning and computer vision (Dell et al. 2014; Mathis and Mathis 2020; Rajagukguk et al. 2025). A wide range of open‐source tools is now available, differing in their tracking strategies and intended applications. Pose‐estimation frameworks such as DeepLabCut (Mathis et al. 2018) and SLEAP (Pereira et al. 2022) estimate the positions of predefined body parts using deep neural networks, whereas identity‐tracking systems such as idTracker.ai (Romero‐Ferrero et al. 2019) focus on maintaining individual identities within large groups. Lightweight and user‐friendly tools, including UMA Tracker (Yamanaka and Takeuchi 2018) and FlyTracker (Eyjolfsdottir et al. 2014), emphasize simplified workflows and lower computational demands. More recent approaches prioritizing real‐time processing and scalability, such as TRex (Walter and Couzin 2021), have further expanded the scope of automated tracking. Collectively, these tools enable large‐scale, high‐resolution trajectory extraction, making them well‐suited for studies requiring high throughput or long‐term monitoring.

Despite their strengths, the adoption of automated tracking tools poses challenges that are distinct from data analysis itself, particularly in exploratory research contexts. Achieving reliable performance typically requires substantial upfront investment: understanding tool‐specific workflows, configuring computational environments, and optimizing recording conditions—lighting, background materials, camera settings—to meet algorithm requirements that often exceed human visual detectability. Moreover, tracking performance varies widely depending on recording conditions, target species, and analytical objectives, making the choice of an appropriate tool highly context dependent and often apparent only after practical evaluation. As a result, users frequently engage in iterative trial‐and‐error processes involving parameter tuning, model selection, and post hoc correction—sometimes across multiple tools—before analyzable data can be obtained. While such investments are justified for large datasets or repeated experiments, they can represent a disproportionate burden for pilot studies, exploratory analyses, or opportunistically recorded videos, where the primary goal is to quickly assess whether a given experimental setup yields biologically meaningful patterns. In these contexts—despite differing end goals—a common requirement emerges: the ability to obtain analyzable trajectory data with minimal learning overhead and rapid turnaround time.

**FIGURE 1:**
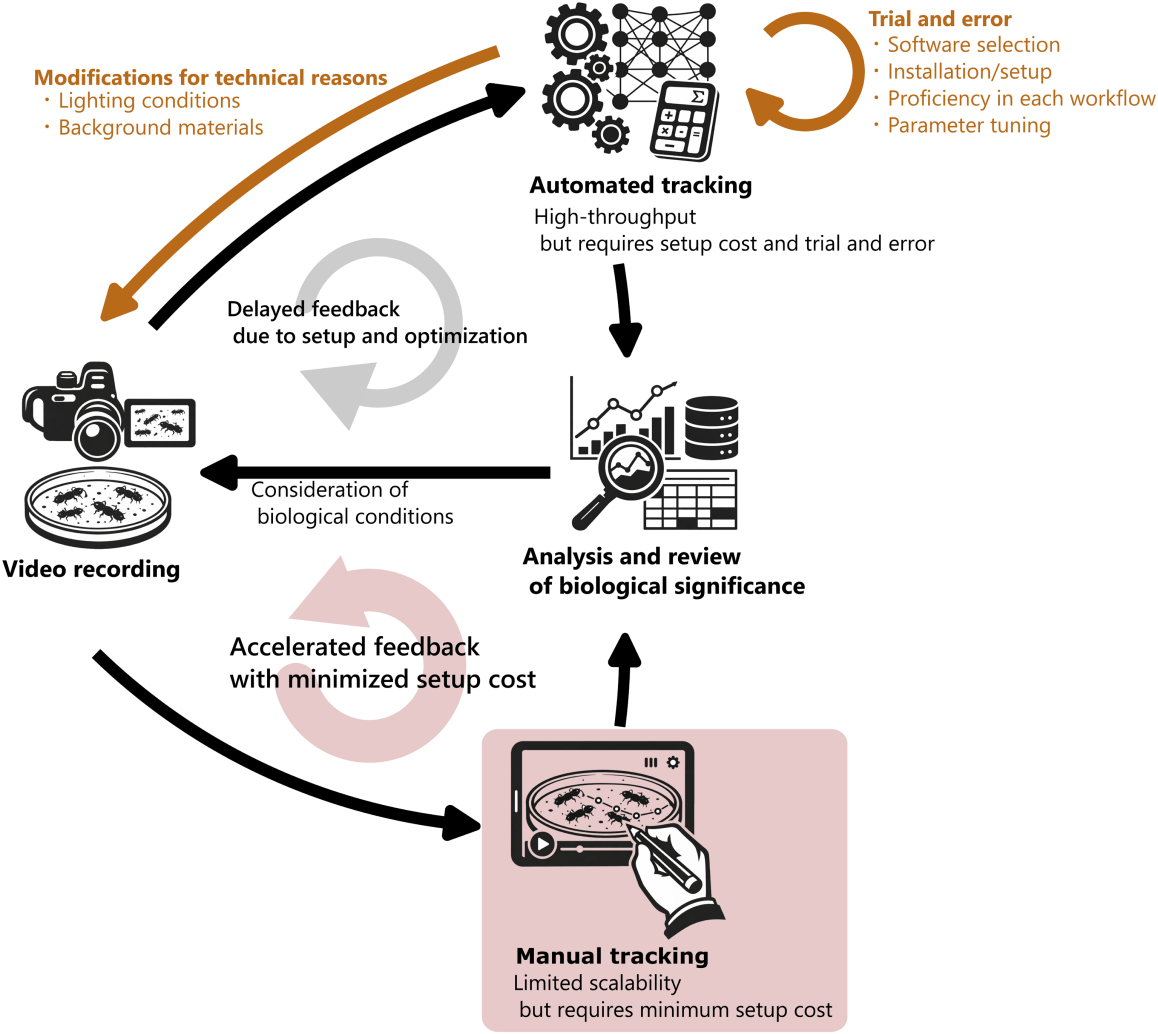
Conceptual comparison of feedback loops in automated and manual video tracking workflows. Automated tracking enables high‐throughput extraction of movement data but typically requires substantial setup or optimization costs. These requirements can delay feedback between video recording and biological interpretation, particularly in exploratory phases. In contrast, manual tracking involves lower throughput but minimal setup cost, allowing rapid feedback between data acquisition and biological evaluation with small data. This accelerated feedback facilitates iterative consideration of experimental and biological conditions during early‐stage or exploratory studies. ManuTrace is positioned within the manual tracking workflow.

In such contexts, the speed and structure of feedback become critical for advancing biological understanding. Behavioral research fundamentally relies on a feedback loop in which biological observations inform experimental design, interpretation, and subsequent refinement of hypotheses (Gomez‐Marin et al. 2014; Tinbergen 1963). However, when automated tracking is used as the sole entry point to trajectory analysis, this biologically driven feedback can be delayed by technically motivated trial‐and‐error cycles required to achieve acceptable tracking performance. As conceptualized in Figure 1, technical optimization—driven by software requirements and recording constraints—and biological evaluation—driven by interpretation of observed behaviors—form distinct feedback loops. In exploratory studies, rapidly iterating the biological feedback loop is often more important than maximizing data throughput, yet automated pipelines—by necessitating extended technical optimization—can delay the biological insights needed to refine experimental design.

Manual tracking remains a common fallback in such situations, but existing software options frequently impose their own limitations. General‐purpose platforms such as ImageJ (Schneider et al. 2012) or custom scripting‐based interfaces often require users to alternate between frame navigation, coordinate measurement, and data management, resulting in fragmented and cognitively demanding workflows. Moreover, many of these tools were not designed specifically for continuous trajectory reconstruction, making it cumbersome to generate smooth, reproducible representations of movement across frames. Some browser‐based annotation platforms also rely on browser–server workflows or server‐side processing, which may introduce delays associated with upload speed or server load and can raise practical concerns about the handling of user‐provided video data. Dedicated desktop applications may also require installation or operating‐system‐specific setup, further restricting accessibility, particularly in educational settings or heterogeneous computing environments. As a result, despite its conceptual simplicity, manual tracking has lacked a dedicated platform optimized for intuitive operation, rapid feedback, and reproducible data output.

To address these challenges, we developed ManuTrace, an interactive, HTML‐based application for manual extraction of trajectory data from videos. ManuTrace allows users to play, pause, and seek videos while recording object positions through mouse clicks, with intermediate positions automatically interpolated to reconstruct continuous trajectories. Because the software consists of a single HTML file, it requires no installation, no external dependencies, and no internet connection and can be executed in any modern web browser. Designed with accessibility, portability, and intuitive operation as guiding principles, ManuTrace enables rapid trajectory extraction under a wide range of recording conditions. In the following sections, we describe the implementation of the software, demonstrate its application using termite behavioral data, and discuss how ManuTrace complements automated tracking tools by facilitating biologically focused, exploratory analyses.

## 2 Software Description

The software is publicly accessible via GitHub Pages (https://yabe‐k.github.io/manutrace/dist/ManuTrace.html) and archived on Zenodo (https://doi.org/10.5281/zenodo.18171002). The GitHub Pages deployment reflects the latest version of the code in the main branch and is provided for convenient hands‐on use, whereas the version archived on Zenodo represents the exact version used in this study.

**FIGURE 2:**
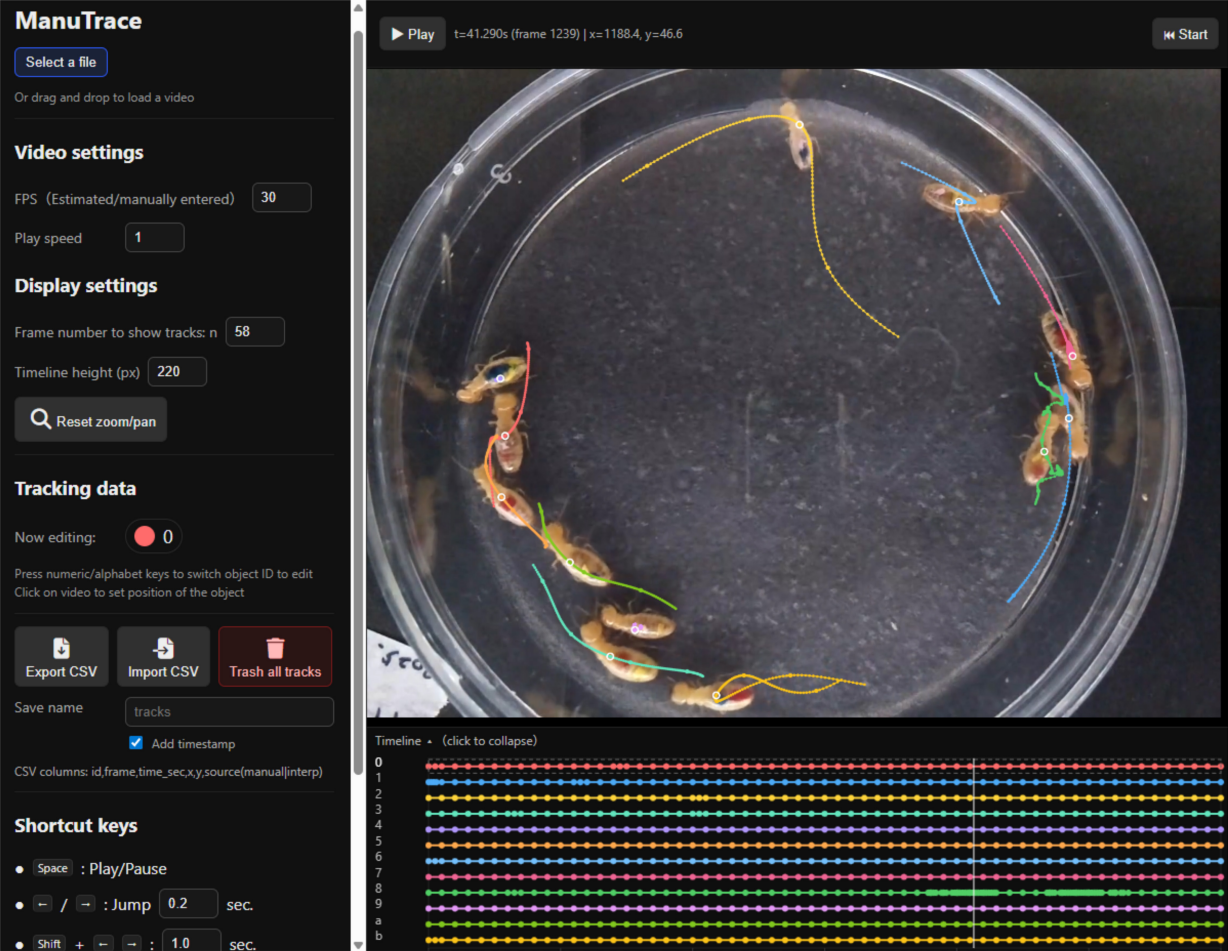
Screenshot of the ManuTrace interface during trajectory annotation. The interface consists of a settings panel on the left, a central video display with overlaid trajectories for multiple individuals, and a timeline at the bottom showing manually recorded positions and interpolated segments for each object. Object positions are specified by mouse clicks on paused video frames, and trajectories are reconstructed by spline interpolation across frames. The example shown illustrates simultaneous tracking of 12 individuals in a termite group video.

ManuTrace is designed for manual tracking of animals in videos, primarily targeting datasets with small to moderate numbers of individuals. It is particularly suitable for behavioral analyses and pilot data collection under conditions in which fully automated tracking is difficult. The user interface is designed to be intuitive, relying mainly on mouse‐based operations (clicking and wheel scrolling) and keyboard shortcuts (Figure 2; Video S1).

ManuTrace is implemented as a single‐file HTML application and can be used without additional installation on any operating system as long as a web browser is available. The software has been tested on Chrome (v.143 on Windows 11 and macOS 26.0.1), Firefox (v.146 on Windows 11), and Safari (26.0.1 on macOS 26.0.1). Because no communication with external servers is required, all data remain local and protected, and the software can be used offline once the HTML file is saved locally. No high‐performance external GPU is required; the software runs smoothly on standard laptops capable of basic video playback.

### 2.1 Control of Video Playback

Analysis begins by loading a video file via drag‐and‐drop into the central area of the screen. Supported video formats depend on the browser, and videos may need to be converted in advance if necessary. In particular, even for MP4 files, which are generally supported, playback may fail depending on the codec used (e.g., codecs such as mp4v are not supported by Chrome, Firefox, or Safari). In such cases, conversion to a supported codec such as H.264 is required. For instance, using ffmpeg, conversion can be performed with a command such as “ffmpeg ‐i $SOURCE_FILENAME ‐c:v libx264 ‐an $OUTPUT_FILENAME.” Video playback can be started and paused using the space key. Zooming is controlled with the mouse wheel, and the view can be panned by right‐click dragging. Seeking through the video is assigned to the left and right arrow keys as well as to Ctrl + mouse wheel operations. The seek step size can be adjusted in the settings panel on the left side of the screen, and modifier keys can be used to further increase or decrease the seek amount. These functions allow users to efficiently record coordinates at fixed temporal intervals if desired.

### 2.2 Recording Trajectories

When the video is paused, clicking on the position of an object in the video frame records its coordinates at that time point. Once coordinates are recorded at multiple time points, positions for all intervening frames are automatically interpolated using cubic spline interpolation, applied separately to the x‐ and y‐coordinates as functions of time. The resulting trajectory is visualized directly on the video. By repeatedly specifying object positions while advancing through the video, users can obtain complete trajectories spanning the entire duration of the video. Position correction follows the same frame‐based interaction: users move to the target frame and update the object position by clicking on the video. This applies to both manually recorded and interpolated points, ensuring a consistent editing workflow. Drag‐and‐drop relocation of points across frames is not supported; instead, any adjustment is performed explicitly at the selected frame.

Up to 36 objects can be registered simultaneously, each assigned an ID corresponding to a digit (0–9) or a lowercase letter (a–z). Switching the active object for recording is performed instantaneously by pressing the corresponding key on the keyboard or by clicking a label in timeline view. The currently active object is displayed in the settings panel on the left side of the screen.

As coordinates are recorded, both the manually recorded time points and the interpolated segments are visualized for each object on a timeline displayed at the bottom of the screen, using points and lines, respectively. This visualization allows users to quickly assess progress and to identify time periods with sparse recording density.

In addition, the timeline interface allows users to edit trajectories by splitting and merging track segments. This enables flexible reassignment of object identities and facilitates the correction and integration of tracklets, for example when individuals cross or become temporarily occluded.

### 2.3 Saving and Exporting Trajectory Data

Trajectory data for all objects can be saved as a CSV file by pressing Ctrl + S or by clicking the corresponding icon in the settings panel. The file is saved to the local download folder and formatted as tidy data, with columns describing object identity, frame number, elapsed time (seconds), x–y coordinates, and the source of each coordinate (manual input or interpolation). This format allows the data to be imported directly into common statistical analysis software. Because manually recorded and interpolated coordinates are explicitly distinguished, users can selectively exclude interpolated values and apply alternative interpolation methods using external software if desired.

Saved CSV files can be reloaded using the CSV import function accessible from the settings panel, enabling users to resume work from a previous session. The CSV export function therefore also serves as a convenient mechanism for backing up intermediate results during trajectory annotation.

## 3 Examples of Application

To demonstrate the robustness and practical utility of ManuTrace under variable experimental conditions, we conducted a simple experiment focusing on inter‐individual interactions in termite groups of two species: *Hodotermopsis sjostedti* and *Zootermopsis nevadensis* . We first analyzed videos recorded under conditions similar to routine laboratory rearing (natural condition). These videos were not originally captured for tracking purposes but were recorded to count behavioral events, and no considerations were made to optimize recording conditions for automated tracking. We evaluated the tracking performance of three existing tools that are considered relatively easy to implement and suitable for tracking multiple individuals—UMA Tracker (Yamanaka and Takeuchi 2018), idTracker.ai (Romero‐Ferrero et al. 2019), and TRex (Walter and Couzin 2021)—and compared their performance with that of ManuTrace.

Because automated tracking performed well in regions with high contrast between the background and individuals, we subsequently recorded additional videos under controlled background conditions (tracker‐optimized condition). Using these videos, we conducted a second performance comparison among the tracking tools.

## 4 Materials and Methods

### 4.1 Video Recording in Natural Condition

For each species ( *Z. nevadensis* and *H. sjostedti* ), six males and six females were randomly selected from laboratory‐maintained colonies to form groups of 12 individuals. Three replicate groups were prepared for each species. To enable sex identification during video analysis, individuals were marked on the dorsal side of the abdomen using enamel paint.

Behavioral arenas consisted of polystyrene Petri dishes (inner diameter: 83 mm). A block of pine wood (38 × 38 × 5 mm, W × D × H) was placed at the center of each dish, and powdered BPC medium (Mitaka et al. 2023) was evenly spread around the wood to a thickness of approximately 2 mm. Termite groups were introduced into the arenas and allowed to acclimate for 1 h at room temperature before recording. Videos were recorded for 1 min at room temperature using a smartphone positioned directly above the arena, capturing the entire Petri dish within the field of view.

### 4.2 Video Recording With Controlled Backgrounds

To create arenas with more uniform backgrounds, the bottom surface of the Petri dish was replaced with filter paper. To enhance contrast between the background and termites, whose body coloration ranges from pale yellow to light brown, the filter paper was immersed in black ink for 5 min and subsequently adjusted to an appropriate moisture level before use.

As in the natural condition, groups of 12 individuals were prepared for each species, with three replicate groups per species. After introduction into the arenas, termite groups were allowed to acclimate for 1 h at room temperature. Videos were then recorded for 1 min at room temperature using a Raspberry Pi Camera Module V2 connected to a Raspberry Pi Model 3 B+, with the camera positioned vertically above the arena.

### 4.3 Simulation of Trajectories and Synthetic Video Generation

To obtain benchmark datasets with known ground‐truth trajectories, we generated synthetic videos of moving agents on complex visual backgrounds. Simulations were implemented in Python using custom scripts.

We simulated the movement of 12 agents in a two‐dimensional continuous space. Each agent followed a stochastic movement process approximating a correlated random walk. At each time step, the position of agent i was updated as:

$$x_{i}\left(t+1\right)=x_{i}\left(t\right)+v_{i}\left(t\right)$$

where $v_{i}\left(t\right)$ is a velocity vector whose direction is drawn from a distribution centered on the previous direction (introducing directional persistence), and whose magnitude is constant. Boundary conditions were implemented as reflective walls to confine agents within the frame.

To create visually challenging conditions for tracking, we generated spatially heterogeneous backgrounds using fractal noise. Specifically, Perlin‐like fractal noise fields were constructed by summing multiple octaves of spatial noise with increasing frequency and decreasing amplitude. This produced naturalistic textures with multi‐scale structure.

Two background conditions were prepared. In the static noise condition, a single fractal noise field was generated and held constant throughout the video. In contrast, in the dynamic noise condition, the fractal noise field was updated over time by introducing a temporal component to the noise function, resulting in smoothly varying background patterns. In this condition, the temporally varying noise was applied to the entire frame, including the regions occupied by agent markers, such that visual contrast between agents and background fluctuated over time.

Agent positions were rendered as circular markers overlaid on the noise background. Each frame was generated sequentially using OpenCV, with fixed spatial resolution and frame rate across conditions. Motion blur and other rendering artifacts were not included, ensuring that all uncertainty arises from background complexity rather than rendering effects. For each simulated video, the true positions of all agents at every frame were recorded and exported as CSV files. These data provide exact trajectories against which tracking outputs can be quantitatively evaluated.

### 4.4 Trajectory Extraction by ManuTrace and Automation Software

For manual tracking using ManuTrace, object positions were recorded at a baseline interval of 0.2 s. To optimize annotation efficiency, the sampling interval was adjusted according to the movement speed of the individuals, with a maximum interval constraint of 1.0 s (i.e., at least one coordinate was recorded per second). Intermediate positions were automatically interpolated using cubic splines.

For automated tracking, we used idTracker.ai (6.0.9), UMA Tracker (Release‐15), and TRex (v2.0.0). Unlike ManuTrace, missing data (NAs) in the trajectories generated by these automated tools were not interpolated and were left as gaps in the output.

In idTracker.ai, the region of interest (ROI) was manually defined, and the number of animals was set to 12. Background subtraction was first applied to detect all individuals; when this approach failed to detect all individuals (6 of the 12 videos), detection based on intensity was instead performed using the “Bright animals” preset. In these cases, blob area thresholds were manually adjusted for each video to match the projected size of the termites. For simulated videos, no ROI was defined because the entire frame constituted the detection area, and background subtraction was used for all videos.

In UMA Tracker, the analysis area was defined using the Circular Selection tool. Individuals were segmented using color filters targeting the specific hues of the dorsal markings or body color (multiple color ranges were combined using the OR logic when necessary). Subsequent “BGR to Gray” conversion was applied, and binarization thresholds were adjusted for each video to optimize segmentation. For simulated videos, Circular Selection was not applied, as the entire frame was used for detection. During color filtering, the color corresponding to the agent markers was directly specified. After binarization, a closing operation (morphological closing) was applied to connect nearby small regions into unified blobs.

In TRex, videos were first converted to grayscale. We used the “background_subtraction” detection mode with a threshold of 50. The maximum number of individuals was set to 12, and the encoding mode was set to “gray.” Spatial scaling was calibrated based on the known diameter of the Petri dish to convert pixels to centimeters. The “track_max_speed” was set to 3 cm/s, and “track_size_filter” was configured with a minimum area ranging from 0.1 to 0.13 cm^2^ (depending on the video) and a maximum of 10 cm^2^. For simulated videos, the detection threshold was set to 15–50, with values determined for each video based on visual inspection of the tracking outputs. Spatial scaling to physical units was not applied, and the “track_max_speed” constraint was not used. Size‐based filtering was applied when necessary, with thresholds set to a minimum of 100 and a maximum of 10,000 pixels^2^.

### 4.5 Performance Evaluation

For empirical videos, no ground‐truth trajectories were available. Therefore, tracking performance was evaluated based on the occurrence of tracking errors (e.g., missed detection and identity swap) rather than positional accuracy.

For simulated videos, where ground‐truth trajectories were known, tracking accuracy was evaluated based on positional deviation from the true coordinates. Because identity swaps occurred frequently in automated tracking outputs, explicit identity matching between detected and ground‐truth trajectories was not performed. Instead, for each detected coordinate at each frame, the Euclidean distance to the nearest ground‐truth coordinate within the same frame was calculated and used as a measure of detection error. Frames with no detected coordinates were treated as missing values (NaN) and excluded from distance‐based summaries.

## 5 Results

### 5.1 Comparison of Tracking Results Across Tools Using Empirical Videos

**FIGURE 3:**
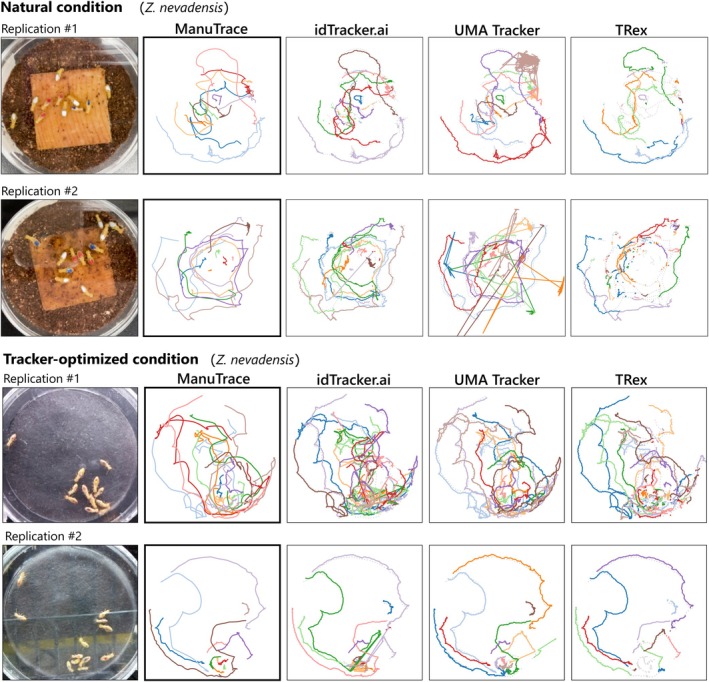
Comparison of tracking trajectories across software tools and recording conditions. Tracking results for groups of *Zootermopsis nevadensis* are shown as representative examples under two recording conditions: Natural conditions and tracker‐optimized conditions. For each condition, two independent replications are presented. Columns show trajectories obtained using ManuTrace and three automated tracking tools (idTracker.ai, UMA Tracker, and TRex). Colored trajectories represent individual identities as assigned by each tracking tool. For automated tools, trajectories generated by ManuTrace are overlaid as light gray dashed lines for reference.

Figure 3 compares tracking results obtained using ManuTrace and three automated tracking tools (idTracker.ai, UMA Tracker, and TRex) across four videos, including two recorded under natural conditions and two recorded under tracker‐optimized conditions. Although some automated tools provide interfaces for manual correction, only the trajectories directly output by each tool are shown here.

The performance of automated tracking tools, assessed in terms of the frequency and type of tracking errors rather than positional accuracy relative to a ground truth, was strongly influenced by recording conditions. For idTracker.ai, the number of error items reported by the software interface ranged from 158 to 857 under natural conditions (mean = 346.5), whereas fewer errors were reported under tracker‐optimized conditions (134–356; mean = 213.0). These values were aggregated from three videos each of *Z. nevadensis* and *H. sjostedti* under both recording conditions. On average, these error levels corresponded to approximately 4 and 2 h of manual correction time, respectively. In UMA Tracker, tracking performance was affected not only by background heterogeneity but also by reflections of light sources on the surface of the Petri dish. Under natural conditions, where lighting was not controlled, misidentification of non‐animal regions occurred frequently (Figure 3, natural condition, replications #1 and #2). Manual correction of these errors required approximately 30 min per individual. In TRex, background subtraction effectively reduced the influence of background complexity. However, substantial variability in tracking performance was observed even when identical parameter settings were applied to videos recorded under the same conditions, indicating that additional tuning was required to achieve stable tracking results.

Individual behavioral characteristics also influenced tracking performance. In the two termite species analyzed, locomotor activity varied widely among individuals. TRex failed to detect individuals with low locomotor activity under both recording conditions, although it was less sensitive to background conditions than the other automated tools. This observation is consistent with characteristics described in the official documentation of TRex. In addition, aggregation of individuals, a common feature of social insects, adversely affected tracking performance across all automated tools, resulting in identity swaps and failures in contour detection that reduced the apparent number of tracked individuals. These issues persisted even under tracker‐optimized conditions, limiting the usability of automated tracking without manual correction.

In contrast, ManuTrace required only that objects be visually identifiable in the video and showed stable performance across recording conditions. For both natural and tracker‐optimized conditions, the time required to extract trajectories of 12 individuals from a 1‐min video (1800 frames) was at most 20 min. This processing time was substantially shorter than the manual correction time required for the automated tracking tools under the conditions tested.

### 5.2 Comparison of Termite Locomotor Activity Under Different Recording Conditions

**FIGURE 4:**
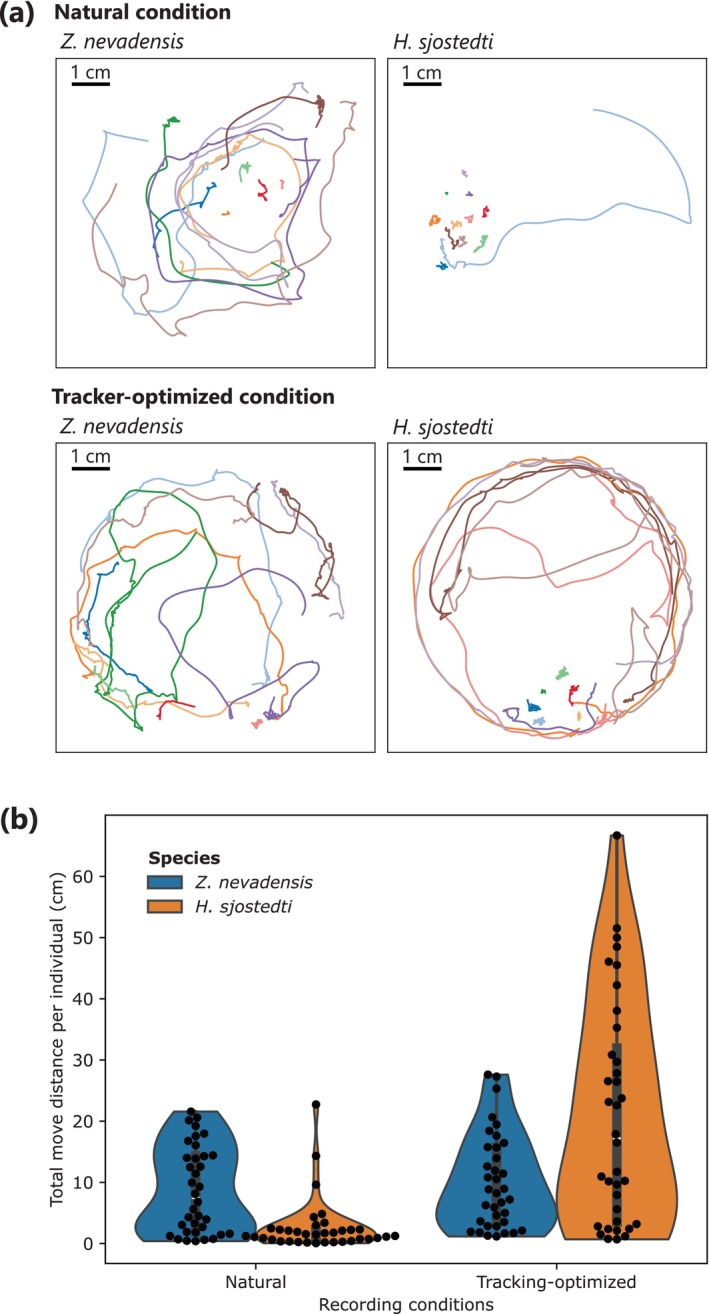
Effects of recording conditions on locomotor activity in two termite species. (a) Representative movement trajectories of *Zootermopsis nevadensis* and *Hodotermopsis sjostedti* recorded under natural conditions and tracker‐optimized conditions. For each species and condition, trajectories from a single video (not overlapping with those shown in Figure 3) are shown as examples. Colored lines indicate trajectories of individual termites. (b) Total distance traveled per individual during a 1‐min video, calculated from trajectories obtained using ManuTrace. Data are pooled across three videos for each species and recording condition. Distributions illustrate differences in locomotor activity between species and between recording conditions, revealing a significant interaction between species and recording condition.

Under natural recording conditions, many individuals of both termite species remained aggregated and showed limited movement, whereas higher locomotor activity was observed under tracker‐optimized conditions (Figure 4a). Using trajectory data obtained with ManuTrace, locomotor activity was quantified for each individual across two species, two recording conditions, three replicate videos per condition, and 12 individuals per video (Figure 4b).

To account for non‐independence among individuals recorded within the same video, locomotor activity was analyzed using a linear mixed‐effects model with species and recording condition as fixed effects and video identity as a random effect. The model revealed a significant main effect of recording condition on total distance traveled (recording condition: *χ* ^2^ = 8.48, df = 1, *p* = 0.00359). Moreover, the interaction between species and recording condition was also significant (species × recording condition: *χ* ^2^ = 9.51, df = 1, *p* = 0.00205), as assessed by likelihood ratio tests comparing nested models with and without each term.

### 5.3 Comparison With Ground‐Truth Trajectories Using Simulated Videos

**FIGURE 5:**
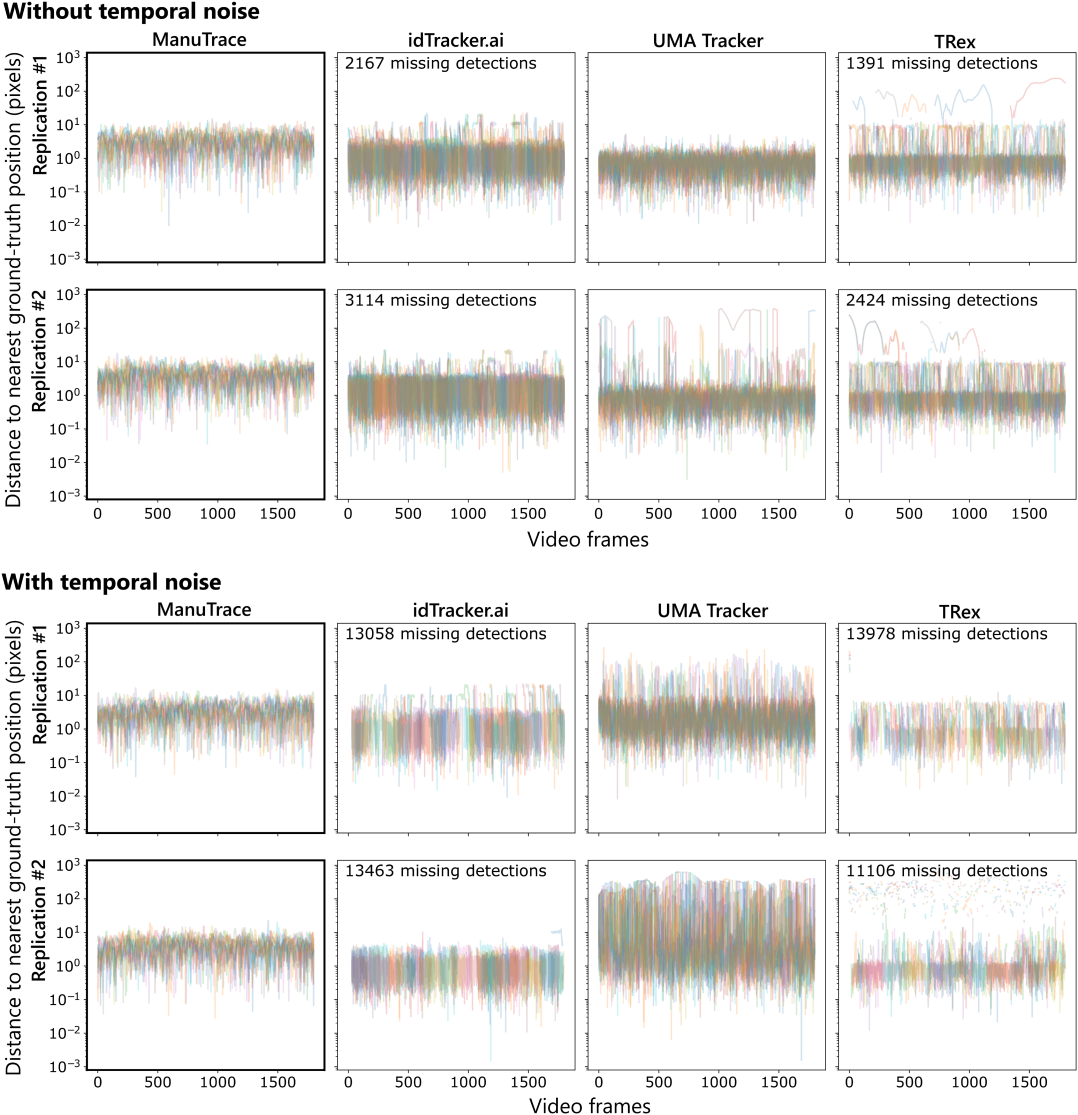
Comparison of tracking accuracy across methods using simulated videos with and without temporally varying noise. Positional error is shown as the frame‐wise nearest‐neighbor distance between detected coordinates and ground‐truth positions (pixels, log scale). Each panel represents one combination of tracking method (columns: ManuTrace, idTracker.ai, UMA Tracker, TRex), noise condition (top: Without temporal noise; bottom: With temporal noise), and replicate (two rows per condition). Colored lines correspond to individual trajectories. Automated tracking methods generally achieved low positional errors when detection was successful but exhibited occasional large deviations and missing detections, particularly under temporally varying noise. Missing detections are indicated by the number of frames without valid detections in each panel. In contrast, ManuTrace produced stable error distributions across conditions, with deviations remaining within a relatively narrow range. The residual error in ManuTrace reflects interpolation between manually annotated frames rather than detection failure.

Tracking accuracy varied substantially among methods and across background conditions (Figure 5). For the automated tracking tools, positional deviations from the ground truth were generally small for correctly detected points, indicating that these methods can achieve high accuracy when detection is successful. However, all automated tools exhibited occasional large deviations, corresponding to incorrect detections or mismatches. Among the automated methods, UMA Tracker showed a distinct error pattern: because the number of detected objects is constrained to a predefined value, the algorithm returned detections even when no appropriate candidates were present. This resulted in frequent large positional errors under conditions where reliable segmentation was difficult.

Background conditions strongly influenced tracking performance. In particular, under temporally varying noise, all automated methods showed a marked increase in both the frequency of large positional deviations and the number of missing detections. This indicates that time‐varying background structure substantially degrades detection stability in automated pipelines.

In contrast, trajectories obtained using ManuTrace were stable across all conditions, with positional deviations remaining within a relatively narrow range regardless of background complexity. This reflects the robustness of manual tracking to visual noise and temporal variation.

However, even under conditions where automated methods performed well, ManuTrace exhibited systematically larger positional deviations, with errors reaching up to approximately 10 pixels. This discrepancy arises because ManuTrace does not record positions at every frame; instead, coordinates between manually annotated frames are interpolated using cubic splines. As a result, interpolation error introduces a baseline level of positional deviation even in the absence of detection failure.

## 6 Discussion

Through comparative evaluation of multiple tracking tools, this study highlights that different tracking approaches entail distinct trade‐offs across recording conditions and behavioral contexts. Automated tracking tools exhibited varying strengths depending on background properties and behavioral characteristics of the target organisms, indicating that efficient data acquisition requires careful matching between recording conditions and tracking algorithms. Comparison with simulated ground‐truth data revealed a fundamental difference in error structure between approaches: automated methods tend to produce low positional errors when detection is successful but are prone to large, sporadic deviations and missing detections, whereas ManuTrace introduces a relatively consistent interpolation‐related bias while avoiding catastrophic tracking failures. Because ManuTrace requires only that individuals are visually identifiable in video frames, it enables stable and predictable trajectory extraction across diverse recording conditions.

Comparison of termite behavior across recording environments further revealed that recording conditions can directly influence individual locomotor activity, and that these effects differ between species. This finding indicates that experimental conditions optimized solely to satisfy technical requirements of tracking algorithms may introduce unintended bias into behavioral measurements. Because ManuTrace provides consistent data quality across contrasting recording environments, it offers a useful means to assess and validate potential behavioral effects of recording conditions themselves. In the present study, the termites in tracker‐optimized videos were sufficiently detectable that conventional computer‐vision‐based tools could recover trajectories under favorable conditions. However, many biologically relevant recording settings—particularly field environments—are characterized by heterogeneous and visually complex backgrounds, occlusions, and variable illumination that can substantially reduce the reliability of automated tracking unless extensive model training or workflow optimization is performed. In such contexts, ManuTrace can facilitate trajectory extraction without requiring substantial modification of the experimental environment.

Although automated tracking tools may achieve improved performance through extensive parameter tuning and post hoc correction, such optimization entails additional time investment. When the number of available videos is limited, the time required for repeated tuning and manual correction may exceed the time savings afforded by automation. Under these circumstances—such as exploratory studies, pilot experiments, or analyses of opportunistically recorded videos—the broad applicability and minimal setup requirements of ManuTrace provide a practical advantage, despite its lower throughput relative to fully automated approaches. In addition, ManuTrace can be used to refine and integrate tracklets generated by automated tracking tools. Because identity tracking often fails under conditions involving occlusions or frequent interactions (Dell et al. 2014; Rajagukguk et al. 2025), the ability to split, merge, and reassign trajectory segments provides a practical means to correct fragmented tracks and improve overall trajectory consistency.

Practical considerations related to software adoption further highlight this distinction. The comparison presented here assumes that automated tracking tools are already properly installed and configured; however, in practice, the initial setup and troubleshooting required to deploy multiple tracking tools can represent a substantial barrier. This burden is amplified when multiple tools must be evaluated to identify an appropriate workflow for a given dataset. In addition, some automated tools require high‐performance computational resources, such as large amounts of RAM or dedicated GPUs, which may limit accessibility. In contrast, ManuTrace requires only a standard web browser and sufficient hardware to play video files, with no installation or internet connection necessary. These low technical requirements substantially reduce barriers to initial data exploration and trial analyses.

In ManuTrace, positions recorded at discrete time points are interpolated using cubic splines to reconstruct continuous trajectories. This is a commonly used approach, but—as with any interpolation method—it necessarily imposes assumptions on unobserved movement. Consequently, its validity may not hold under all behavioral patterns, particularly in cases of rapid or highly discontinuous motion (Long 2016; Tremblay et al. 2006). In the present study, however, comparison with ground‐truth trajectories in simulated data showed that interpolation‐induced deviations remained relatively small and stable, within approximately 10 pixels under the tested conditions. This error reflects interpolation uncertainty rather than detection failure and should therefore be interpreted differently from the large, sporadic deviations observed in automated tracking outputs. In addition, ManuTrace enables users to annotate all frames, when higher accuracy is required. This enables quantitative evaluation of interpolation accuracy, for example by subsampling fully annotated trajectories at fixed intervals, applying cubic spline interpolation, and quantitatively comparing the reconstructed trajectories with the original data to assess the impact of sampling interval. Importantly, the adequacy of interpolation depends on the downstream analysis: while coarse metrics such as total trajectory length may be relatively robust, quantities sensitive to high‐frequency variation, such as instantaneous velocity or turning frequency, may be more strongly affected (Codling and Hill 2005; Rosser et al. 2013). In situations where interpolation assumptions are violated, the spline function should therefore be regarded primarily as a visual aid for trajectory construction rather than as a definitive source of quantitative estimates.

More generally, the advantages of ManuTrace must be considered alongside its limitations. Because tracking is not fully automated, annotating long videos or datasets containing large numbers of individuals can be time‐consuming, and the software is not intended to replace high‐throughput automated tracking pipelines. Manual annotation may also introduce observer‐dependent variation. However, temporal resolution can be partially standardized by recording positions at fixed time intervals using predefined seek steps, allowing users to exert quantitative control over temporal sampling when required. In addition, ManuTrace is limited to two‐dimensional tracking based on image‐plane coordinates and does not capture depth or three‐dimensional posture, which restricts its applicability to analyses focused on planar movement patterns.

Taken together, our results position ManuTrace not as an alternative to automated tracking tools, but as a complementary approach optimized for exploratory and small‐scale analyses. By minimizing setup cost and technical barriers, ManuTrace enables rapid feedback between video recording, trajectory extraction, and biological interpretation. This accelerated feedback is particularly valuable during early stages of experimental design, when recording conditions and analytical goals are still being refined. In combination with automated methods, ManuTrace provides a flexible and accessible entry point to trajectory analysis, supporting robust behavioral inference while preserving experimental conditions that reflect natural biological contexts.

## Author Contributions

**Kiyotaka Yabe:** conceptualization (lead), data curation (equal), formal analysis (lead), funding acquisition (supporting), investigation (supporting), methodology (equal), project administration (equal), resources (supporting), software (lead), validation (equal), visualization (lead), writing – original draft (lead). **Yuna Tosaka:** conceptualization (supporting), data curation (equal), formal analysis (supporting), funding acquisition (supporting), investigation (lead), methodology (equal), resources (lead), software (supporting), validation (equal), visualization (supporting), writing – original draft (supporting), writing – review and editing (equal). **Kenji Matsuura:** funding acquisition (lead), project administration (equal), resources (supporting), supervision (lead), writing – review and editing (equal).

## Funding

This work was supported by the JSPS KAKENHI Grant Numbers JP23H00332 (to K.M.) and JP24KJ1453 (to K.Y.), the Cabinet Office, Government of Japan, Moonshot R&D Program for Agriculture, Forestry and Fisheries (funding agency: Bio‐oriented Technology Research Advancement Institution) Project # JPJ009237 (to K.M.), and the Distinguished Doctoral Program of Platforms, Kyoto University (WISE) (to K.Y. and Y.T.).

## Conflicts of Interest

The authors declare no conflicts of interest.

## Supporting information


**Video S1:** Demonstration of a typical manual tracking workflow in ManuTrace. The video illustrates video navigation, object selection, position annotation, and trajectory visualization during trajectory extraction from animal movement videos.

## Data Availability

All data and code necessary to reproduce the results of this study are publicly available. The complete dataset—including all video files analyzed in the manuscript, trajectory outputs generated by ManuTrace and by the automated tracking tools evaluated in this study, and the scripts used for statistical analysis and figure generation—is provided in the public GitHub repository for the ManuTrace software. The exact version corresponding to this study has been archived on Zenodo and assigned a permanent DOI (https://doi.org/10.5281/zenodo.19946363), ensuring long‐term accessibility and reproducibility.

## References

[ece373804-bib-0001] Anderson, D. J. , and P. Perona . 2014. “Toward a Science of Computational Ethology.” Neuron 84, no. 1: 18–31. 10.1016/J.NEURON.2014.09.005. 25277452

[ece373804-bib-0002] Ballerini, M. , N. Cabibbo , R. Candelier , et al. 2008. “Interaction Ruling Animal Collective Behavior Depends on Topological Rather Than Metric Distance: Evidence From a Field Study.” Proceedings of the National Academy of Sciences 105, no. 4: 1232–1237. 10.1073/PNAS.0711437105. PMC2234121 18227508

[ece373804-bib-0003] Bell, W. J. 1990. Searching Behaviour. Springer Netherlands. 10.1007/978-94-011-3098-1.

[ece373804-bib-0004] Benhamou, S. 2004. “How to Reliably Estimate the Tortuosity of an Animal's Path: Straightness, Sinuosity, or Fractal Dimension?” Journal of Theoretical Biology 229, no. 2: 209–220. 10.1016/J.JTBI.2004.03.016. 15207476

[ece373804-bib-0005] Brown, A. E. X. , and B. De Bivort . 2018. “Ethology as a Physical Science.” Nature Physics 14, no. 7: 653–657. 10.1038/s41567-018-0093-0.

[ece373804-bib-0006] Buhl, J. , D. J. T. Sumpter , I. D. Couzin , et al. 2006. “From Disorder to Order in Marching Locusts.” Science 312, no. 5778: 1402–1406. 10.1126/science.1125142. 16741126

[ece373804-bib-0007] Codling, E. A. , and N. A. Hill . 2005. “Sampling Rate Effects on Measurements of Correlated and Biased Random Walks.” Journal of Theoretical Biology 233, no. 4: 573–588. 10.1016/J.JTBI.2004.11.008. 15748917

[ece373804-bib-0008] Couzin, I. D. , J. Krause , N. R. Franks , and S. A. Levin . 2005. “Effective Leadership and Decision‐Making in Animal Groups on the Move.” Nature 433, no. 7025: 513–516. 10.1038/nature03236. 15690039

[ece373804-bib-0009] Dell, A. I. , J. A. Bender , K. Branson , et al. 2014. “Automated Image‐Based Tracking and Its Application in Ecology.” Trends in Ecology & Evolution 29, no. 7: 417–428. 10.1016/J.TREE.2014.05.004. 24908439

[ece373804-bib-0010] Denenberg, V. H. 1969. “Open‐Field Behavior in the Rat: What Does It Mean?” Annals of the New York Academy of Sciences 159, no. 3: 852–859. 10.1111/j.1749-6632.1969.tb12983.x. 5260302

[ece373804-bib-0011] Dill, L. M. , and R. Houtman . 1989. “The Influence of Distance to Refuge on Flight Initiation Distance in the Gray Squirrel ( *Sciurus carolinensis* ).” Canadian Journal of Zoology 67, no. 1: 233–235. 10.1139/z89-033.

[ece373804-bib-0012] Eyjolfsdottir, E. , S. Branson , X. P. Burgos‐Artizzu , et al. 2014. “Detecting Social Actions of Fruit Flies.” In Computer Vision – ECCV 2014, edited by D. Fleet, T. Pajdla, B. Schiele, and T. Tuytelaars, 772–787. Lecture Notes in Computer Science 8690. Springer. 10.1007/978-3-319-10605-2_50.

[ece373804-bib-0013] Gomez‐Marin, A. , J. J. Paton , A. R. Kampff , R. M. Costa , and Z. F. Mainen . 2014. “Big Behavioral Data: Psychology, Ethology and the Foundations of Neuroscience.” Nature Neuroscience 17, no. 11: 1455–1462. 10.1038/nn.3812. 25349912

[ece373804-bib-0014] Kareiva, P. M. , and N. Shigesada . 1983. “Analyzing Insect Movement as a Correlated Random Walk.” Oecologia 56, no. 2–3: 234–238. 10.1007/BF00379695/METRICS. 28310199

[ece373804-bib-0015] Lima, S. L. , and L. M. Dill . 1990. “Behavioral Decisions Made Under the Risk of Predation: A Review and Prospectus.” Canadian Journal of Zoology 68, no. 4: 619–640. 10.1139/z90-092.

[ece373804-bib-0016] Long, J. A. 2016. “Kinematic Interpolation of Movement Data.” International Journal of Geographical Information Science 30, no. 5: 854–868. 10.1080/13658816.2015.1081909.

[ece373804-bib-0017] Mathis, A. , P. Mamidanna , K. M. Cury , et al. 2018. “DeepLabCut: Markerless Pose Estimation of User‐Defined Body Parts With Deep Learning.” Nature Neuroscience 21, no. 9: 1281–1289. 10.1038/s41593-018-0209-y. 30127430

[ece373804-bib-0018] Mathis, M. W. , and A. Mathis . 2020. “Deep Learning Tools for the Measurement of Animal Behavior in Neuroscience.” Current Opinion in Neurobiology 60: 1–11. 10.1016/J.CONB.2019.10.008. 31791006

[ece373804-bib-0019] Mitaka, Y. , T. Akino , and K. Matsuura . 2023. “Development of a Standard Medium for Culturing the Termite Reticulitermes Speratus.” Insectes Sociaux 70, no. 2: 265–274. 10.1007/s00040-023-00907-6.

[ece373804-bib-0020] Partridge, B. L. 1982. “The Structure and Function of Fish Schools.” Scientific American 246, no. 6: 114–123. http://www.jstor.org/stable/24966618. 7201674 10.1038/scientificamerican0682-114

[ece373804-bib-0021] Pereira, T. D. , N. Tabris , A. Matsliah , et al. 2022. “SLEAP: A Deep Learning System for Multi‐Animal Pose Tracking.” Nature Methods 19, no. 4: 486–495. 10.1038/s41592-022-01426-1. 35379947 PMC9007740

[ece373804-bib-0022] Pyke, G. H. , H. R. Pulliam , and E. L. Charnov . 1977. “Optimal Foraging: A Selective Review of Theory and Tests.” Quarterly Review of Biology 52, no. 2: 137–154. http://www.jstor.org/stable/2824020.

[ece373804-bib-0023] Rajagukguk, R. A. , S. Lee , J. Park , et al. 2025. “Deep Learning for Visual Animal Monitoring (Detection, Tracking, Pose Estimation, and Behavior Classification): A Comprehensive Review.” Smart Agricultural Technology 12: 101539. 10.1016/J.ATECH.2025.101539.

[ece373804-bib-0024] Romero‐Ferrero, F. , M. G. Bergomi , R. C. Hinz , F. J. H. Heras , and G. G. de Polavieja . 2019. “Idtracker.Ai: Tracking All Individuals in Small or Large Collectives of Unmarked Animals.” Nature Methods 16, no. 2: 179–182. 10.1038/s41592-018-0295-5. 30643215

[ece373804-bib-0025] Rosser, G. , A. G. Fletcher , P. K. Maini , and R. E. Baker . 2013. “The Effect of Sampling Rate on Observed Statistics in a Correlated Random Walk.” Journal of the Royal Society Interface 10, no. 85: 20130273. 10.1098/RSIF.2013.0273. 23740484 PMC4043159

[ece373804-bib-0026] Schneider, C. A. , W. S. Rasband , and K. W. Eliceiri . 2012. “NIH Image to ImageJ: 25 Years of Image Analysis.” Nature Methods 9, no. 7: 671–675. 10.1038/nmeth.2089. 22930834 PMC5554542

[ece373804-bib-0027] Tinbergen, N. 1963. “On Aims and Methods of Ethology.” Zeitschrift für Tierpsychologie 20, no. 4: 410–433. 10.1111/j.1439-0310.1963.tb01161.x.

[ece373804-bib-0028] Tremblay, Y. , S. A. Shaffer , S. L. Fowler , et al. 2006. “Interpolation of Animal Tracking Data in a Fluid Environment.” Journal of Experimental Biology 209, no. 1: 128–140. 10.1242/JEB.01970. 16354784

[ece373804-bib-0029] Walter, T. , and I. D. Couzin . 2021. “TRex, a Fast Multi‐Animal Tracking System With Markerless Identification, and 2D Estimation of Posture and Visual Fields.” eLife 10: e64000. 10.7554/eLife.64000. 33634789 PMC8096434

[ece373804-bib-0030] Yamanaka, O. , and R. Takeuchi . 2018. “UMATracker: An Intuitive Image‐Based Tracking Platform.” Journal of Experimental Biology 221: jeb182469. 10.1242/jeb.182469. 29954834

